# Quantum Machine-Based Decision Support System for the Detection of Schizophrenia from EEG Records

**DOI:** 10.1007/s10916-024-02048-0

**Published:** 2024-03-05

**Authors:** Gamzepelin Aksoy, Grégoire Cattan, Subrata Chakraborty, Murat Karabatak

**Affiliations:** 1 Department of Software Engineering, Firat University, Elazig, Türkiye; 2 IBM, Data and AI, Kraków, Poland; 3 School of Science and Technology, Faculty of Science, Agriculture, Business and Law, University of New England, Armidale, NSW 2351 Australia; 4 Centre for Advanced Modelling and Geospatial Information Systems (CAMGIS), School of Civil and Environmental Engineering, Faculty of Engineering and IT, University of Technology Sydney, Ultimo, NSW 2007 Australia; 5 Griffith Business School, Griffith University, Brisbane, QLD 4111 Australia

**Keywords:** Electroencephalography (EEG), Machine Learning (ML), Feature map, Quantum Support Vector Machine (QSVM)

## Abstract

Schizophrenia is a serious chronic mental disorder that significantly affects daily life. Electroencephalography (EEG), a method used to measure mental activities in the brain, is among the techniques employed in the diagnosis of schizophrenia. The symptoms of the disease typically begin in childhood and become more pronounced as one grows older. However, it can be managed with specific treatments. Computer-aided methods can be used to achieve an early diagnosis of this illness. In this study, various machine learning algorithms and the emerging technology of quantum-based machine learning algorithm were used to detect schizophrenia using EEG signals. The principal component analysis (PCA) method was applied to process the obtained data in quantum systems. The data, which were reduced in dimensionality, were transformed into qubit form using various feature maps and provided as input to the Quantum Support Vector Machine (QSVM) algorithm. Thus, the QSVM algorithm was applied using different qubit numbers and different circuits in addition to classical machine learning algorithms. All analyses were conducted in the simulator environment of the IBM Quantum Platform. In the classification of this EEG dataset, it is evident that the QSVM algorithm demonstrated superior performance with a 100% success rate when using Pauli X and Pauli Z feature maps. This study serves as proof that quantum machine learning algorithms can be effectively utilized in the field of healthcare.

## Introduction

Schizophrenia is an exceptionally severe neuropsychological disorder characterized by symptoms such as hallucinations, communication and thought disturbances, abnormal motor behavior, delusions, and disruptions in daily activities. While the precise cause remains unknown, factors like genetic inheritance and environmental influences (migration, childhood traumas, infectious diseases, drug use, nutrition, toxins, urbanization, etc.) play a role in the development of the illness [1, 2]. The incidence of the disease, which typically begins in young adulthood, is similar in most countries. This condition, affecting approximately 1% of the world's population, is more commonly observed in men than women [3]. If schizophrenia is left untreated, the incidence of various complications such as depression, associability, inability to control behavior, tendency to violence, anxiety disorders, and suicide increase. Compared to the general population, people with schizophrenia live 10–25 years less [4]. 4.9% of individuals with the disease choose death by suicide. The cost of the disease is significantly higher compared to other illnesses due to the required healthcare services, social needs, and decreased employment [5]. In these diseases that require lifelong treatment, early diagnosis allows symptoms to be kept under control.

The rapid advancements in technology have led to the emergence of a range of medical imaging techniques that facilitate early diagnosis of diseases. Methods such as Electroencephalography (EEG), Electrocardiography (ECG), Computed Tomography (CT), and Magnetic Resonance Imaging (MRI) have contributed significantly to advancements in the healthcare sector. Among these imaging techniques, Magnetic Resonance Imaging (MRI) and Electroencephalography (EEG) stand out as effective tools, particularly in facilitating the diagnosis process of schizophrenia. EEG records the electrical signal that enables the transmission of information generated by the pyramidal cells through electrodes placed on the scalp [6]. The electrical activity of the brain exhibits complex behaviors characterized by strong non-linear and dynamic properties. Therefore, it is difficult to extract information from EEG signals through observation alone. Various machine learning methods are employed in the data collection process of these signals to extract meaningful information [7].

Quantum-based machine learning algorithms adopt a complex and innovative approach. Going beyond the boundaries of classical algorithms, these methods tackle problems by harnessing the parallel processing, superposition, entanglement, and interference capabilities of quantum computers. The synergy between deep learning's capacity to analyze large datasets and the advent of quantum computing has accelerated technological advancements in the field of data science [8]. Presently, applications of machine learning techniques and deep learning models are frequently encountered in the healthcare sector.

In this study, the goal was to use EEG signals for schizophrenia diagnosis by employing both classical machine learning algorithms and quantum machine learning algorithms. For this purpose, four channels were selected from EEG data obtained from 16 channels, representing different regions. These signals underwent specific preprocessing steps. Principal component analysis was used to reduce the dimensionality of the dataset to 3–15 features. Classical machine learning methods and a quantum support vector machine with different feature maps were applied, and their performances were evaluated.

The contributions of this study to the field can be summarized as follows:

- To the authors' knowledge, this study is the first to use QSVM on EEG datasets for schizophrenia detection.
- The selection of channels that produce the best results from EEG channels has been provided.
- Detailed results of different numbers of qubits generated using the PCA method have been provided for the QSVM algorithm.
- The main feature maps used in quantum machine learning have been compared for their effects on classification. The results suggest that first-order Pauli Map, such as X and Z feature maps provide the best accuracy with EEG data.
- The performance of the QSVM algorithm has been compared with classical machine learning methods.
- This research paper has yielded promising results regarding the use of quantum machine learning in the field of healthcare. Indeed, quantum classifiers are not only able to generalize on EEG data, but they also achieve similar, or slightly better results than the state of the art with a limited number of components.
- The current work suggests that quantum classifiers can be used as a complementary approach to classical technics and help with the diagnosis of schizophrenia.

## Related works

### Classical machine learning techniques for schizophrenia

In the field of schizophrenia, both statistical and deep machine learning models are popular. Statistical machine learning model include Decision Trees (DT), Support Vector Machines (SVM), Logistic Regression (LR), k-Nearest Neighbors (kNN) or ensemble methods such as Random Forest (RF), XGboost or Ensemble Bagged Tree (EBT) [9–18].

For example, in a study involving EEG signals obtained from 256 channels, signals from 8 different regions were processed using a 6th-level wavelet transform. A total of 480 features were extracted, with 12 statistical features applied to each frequency band and region. Classification was conducted on this dataset using the kernel-SVM algorithm, resulting in an accuracy rate of 78.9% [16]. The suitability of kernel-SVM algorithm for the diagnosis of schizophrenia was further confirmed by Sharma et al. [14]. Sharma et al. used 19-channel EEG signals in a study involving 32 healthy individuals and 49 individuals with schizophrenia. Eight machine learning technics, including DT, SVM, RF, and XGBoost were evaluated. According to the study results, it was stated that the SVM algorithm can be used in the diagnosis of schizophrenia with a 100% success rate.

Deep learning model are based mostly on CNN and LSTM, which are popular neural network architectures in the domain of time series analysis [16, 19–25].

For example, A study involving raw EEG data from 101 subjects with 64 channels applied deep learning models, CNN (75.9%), and CNN-LSTM (71.5%). Researchers noted that in terms of classification performance, the T8 and C3 channels, as well as the delta and gamma frequency bands, had a significant impact [23].

In another study where raw EEG data was used in the diagnosis of Alzheimer’s disease and schizophrenia, AC, Grad-CAM, and Saliency Map, which are among the methods of the CNN model, were preferred. A 98% success rate has been achieved using these methods [25]. Deep learning models and hybrid approaches, when provided with sufficient data and proper training, can yield impressive results.

### Quantum machine learning techniques applied to healthcare or EEG

Quantum-based machine learning methods are beginning to find applications in the healthcare sector. However, there are currently no specific studies in the literature focused on schizophrenia diagnosis in the field of quantum machine learning. Nevertheless, access to experimental studies conducted for the diagnosis and treatment of various diseases is possible.

For instance, Yu used the Variational Quantum Classifier (VQC) in PennyLane (Xanadu, Toronto, Canada), a cross-platform Python library, for the analysis of SARS-CoV-2 diseases. The method employed was emphasized as potentially beneficial for early disease diagnosis [26].

In another study, the Quantum Distance Classifier (qDS) and quantum enhanced SVM methods were employed on three different clinical datasets (heart failure, Wisconsin breast cancer, and pediatric bone marrow transplantation). In this study, it was found that the quantum SVM, implemented with only 16 qubits on IBMQ Melbourne, exhibited higher performance [27]. The excellence of the quantum SVM was also confirmed for the classification of diabetes [28] and heart diseases [29].

Kumar et al. conducted a study in which they utilized a quantum version of the kNN and Bayesian classifiers such as DT or RF. They stated that quantum-based methods outperformed traditional methods, with the highest performance achieved by the quantum RF, reaching an accuracy rate of 89% [30].

Shahwar et al. conducted a study presenting a hybrid classical-quantum machine learning model for the detection of Alzheimer's disease. In this study, the complexity and dimension of the data were addressed using hybrid classical-quantum transfer learning. Features extracted by classical neural networks were integrated with a quantum processor. Features were extracted from images using ResNet 34, and feature vectors were generated with QVC. The model was verified using various quantum simulators and achieved a classification accuracy of 97.2% [31].

Padma and Sahoo applied the QKNN, QSVM, and VQC methods to the SWELL KW dataset to examine the effects of stress on mental health. They performed dimensionality reduction by applying Principal Component Analysis (PCA) and Quantum Principal Component Analysis (QPCA) to the data obtained from 25 participants. As a result of the research, they noted that the highest accuracy value was obtained from the QSVM algorithm [32].

At the inverse, in a study aimed at developing a Brain-Computer interface Andreev et al. [33] compared the performance of QSVM with Linear Discriminant Analysis (LDA) in classifying EEG data. This data set, which includes EEG data of 25 people, was obtained using 16 channels in an experiment carried out in the GIPSA laboratory [34]. The experiment was based on the oddball paradigm, which is a common experimental setup for the classification of event-related brain potentials. Brain-Computer interfaces may rely on different experimental paradigms, such as steady-state-visually-evoked potentials or motor imagination (e.g., [35]). These paradigms offer different trade-off between signal-to-noise ration and user comfort. Andreev et al. prepared the data for EEG analysis using the Riemannian Geometry framework and noted that the quantum classifier generalizes well on the EEG data—although the accuracy was lower compared to the analyses conducted with LDA.

When examining these studies, it can be seen that the data sets used and the preprocessing and classifiers applied to these data sets are different. For this reason, performance differences arising from signal-to-noise ratios are observed in all studies. Despite these changes, it is noteworthy that most of the machine learning studies are effective in the field of health – in the sense that they can all generalize from the data and achieve similar or better results than the state of the art.

## Materials and methods

**Fig. 1:**
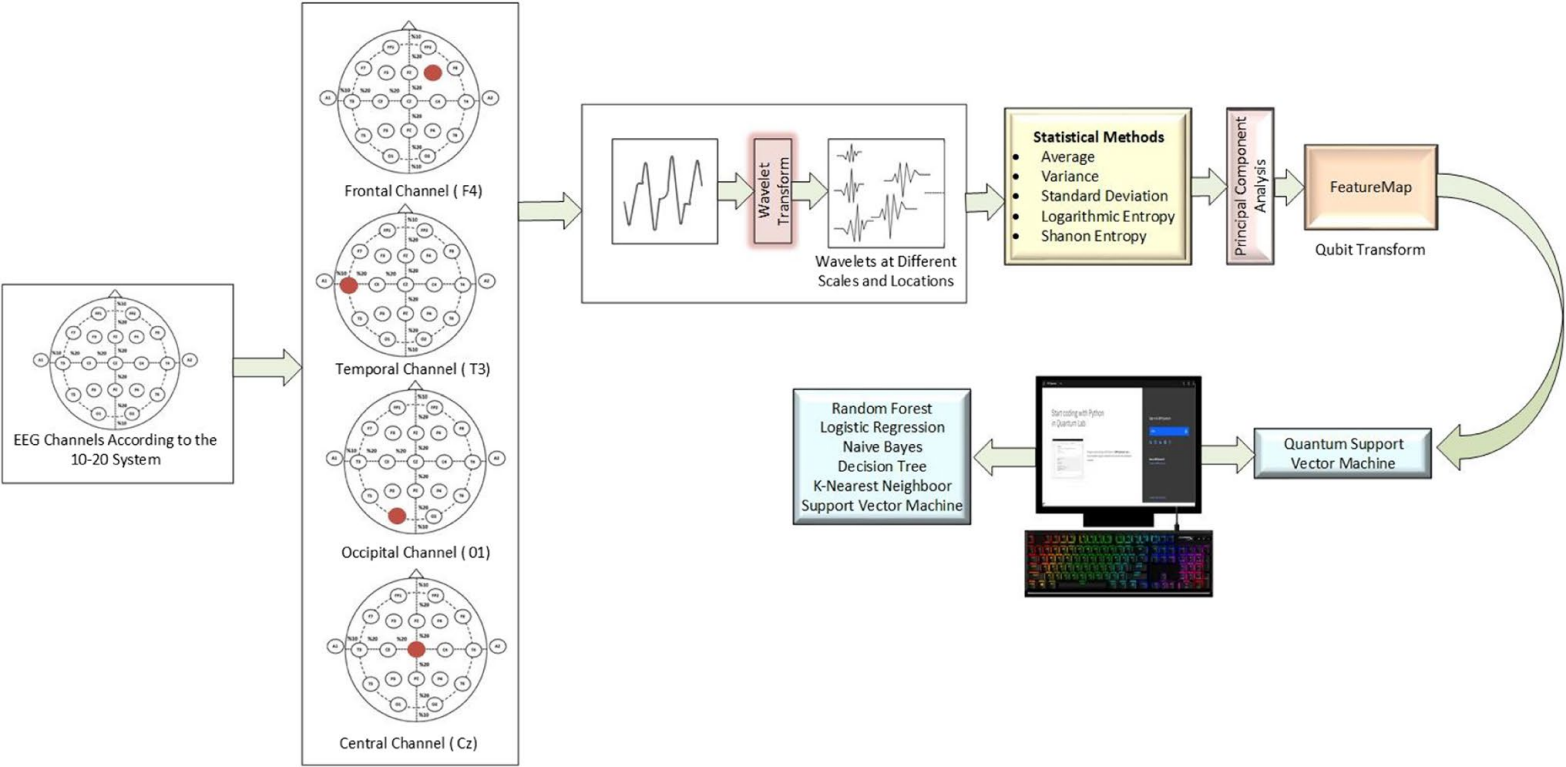
Schematic representation of the method

In this study, an EEG schizophrenia dataset was used to assess the performance of the quantum-based SVM algorithm in detecting the disease [36]. The pattern recognition process is carried out in four stages, which include data acquisition, preprocessing steps, feature extraction, and classification. In the first stage, data acquisition involves discrete wavelet transforms, information measurement methods, and statistical techniques. Subsequently, dimension reduction methods like PCA are employed to prepare the data for analysis in different dimensions. Prepared data is subjected to classical classification algorithms in the IBM Quantum Lab environment. Reduced dimension data is transformed from bit format to qubit format using different feature maps, and quantum-based machine learning models are applied. The performances of classical and quantum-based models are analyzed in terms of accuracy and time, and their suitability for EEG signals is examined. The block diagram of the conducted study is presented in Fig. 1.

### EEG dataset

**Fig. 2:**
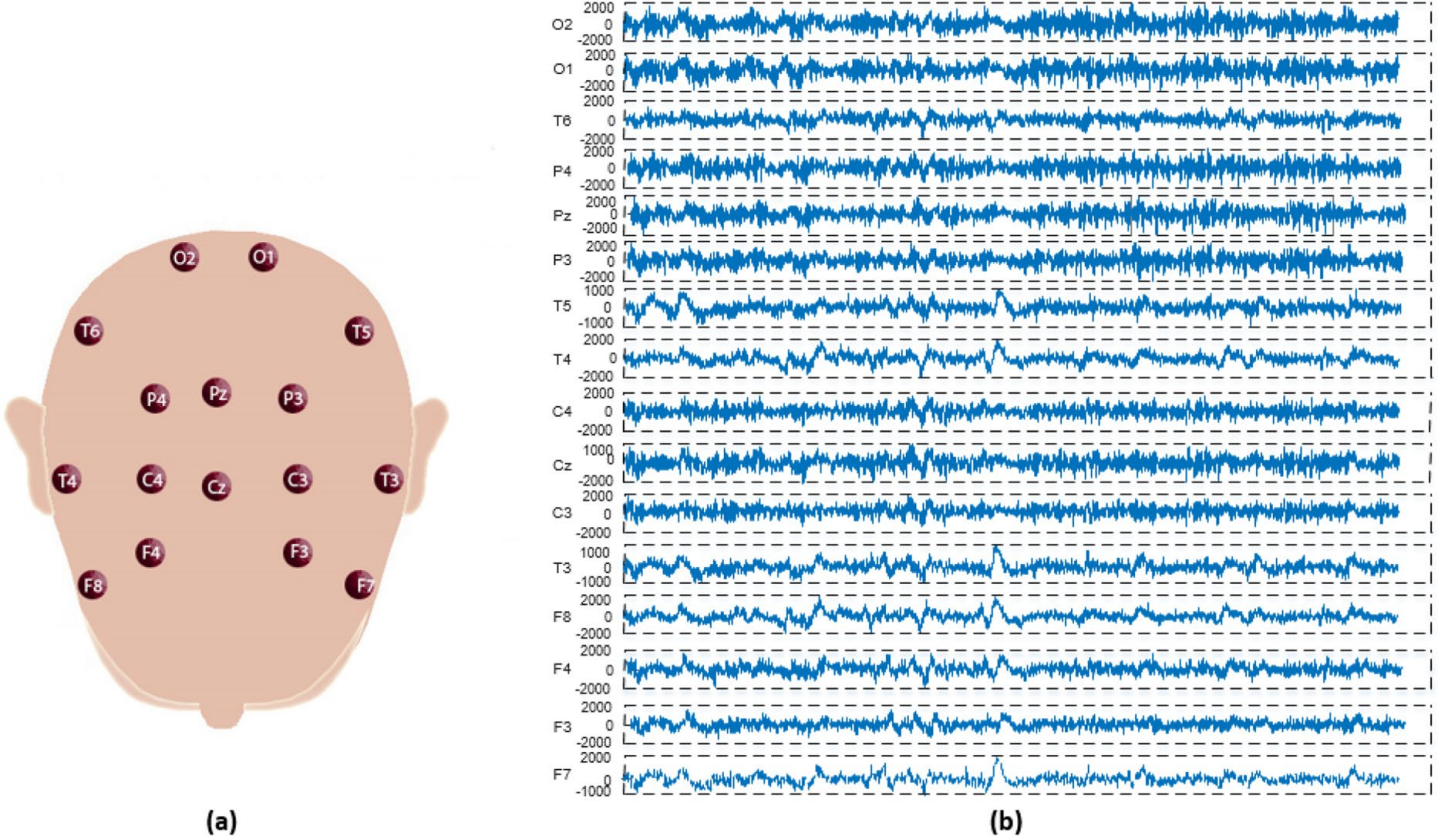
**a** Channels in the EEG dataset. **b** EEG records of a patient individual in the data set

This study utilized a dataset comprising EEG signals collected from 39 healthy individuals and 45 adolescent males. The diagnosis of the affected individuals (childhood schizophrenia, schizophrenic, and schizoaffective disorders) was determined by expert doctors working at the Mental Health Research Center (MHRC). The ages of individuals diagnosed with the disease ranged from 10 years 8 months to 14 years, while the ages of healthy individuals ranged from 11 to 13 years 9 months. The average age range for adolescents in both the patient and control groups is 12 years and 3 months. EEG signals were obtained from 16 channels based on the 10–20 system for electrode placement (O1, O2, P3, P4, Pz, T5, T6, C3, C4, Cz, T3, T4, F3, F4, F7 and F8). It was indicated that individuals had their eyes closed, were awake, and were in a relaxed position during recordings. Artificial EEG segments were not used in this study, and the recordings were made at a sampling rate of 128 Hz. This dataset was obtained by recording 1-min segments from each channel for 84 adolescent individuals [37]. The channels from which the dataset was obtained and the EEG signal data from a patient are shown in Fig. 2.

### Feature extraction

In the dataset containing EEG signals obtained from 16 channels placed on the skulls of schizophrenic and healthy adolescent individuals, four channels from different regions were selected. These channels are F4 from the frontal region, T3 from the temporal region, O1 from the occipital region, and Cz from the central region. A discrete wavelet transform was applied to these selected channels. Subsequently, various statistical methods were employed on the obtained signals to prepare the dataset for analysis.

#### Discrete wavelet transform

**Fig. 3:**
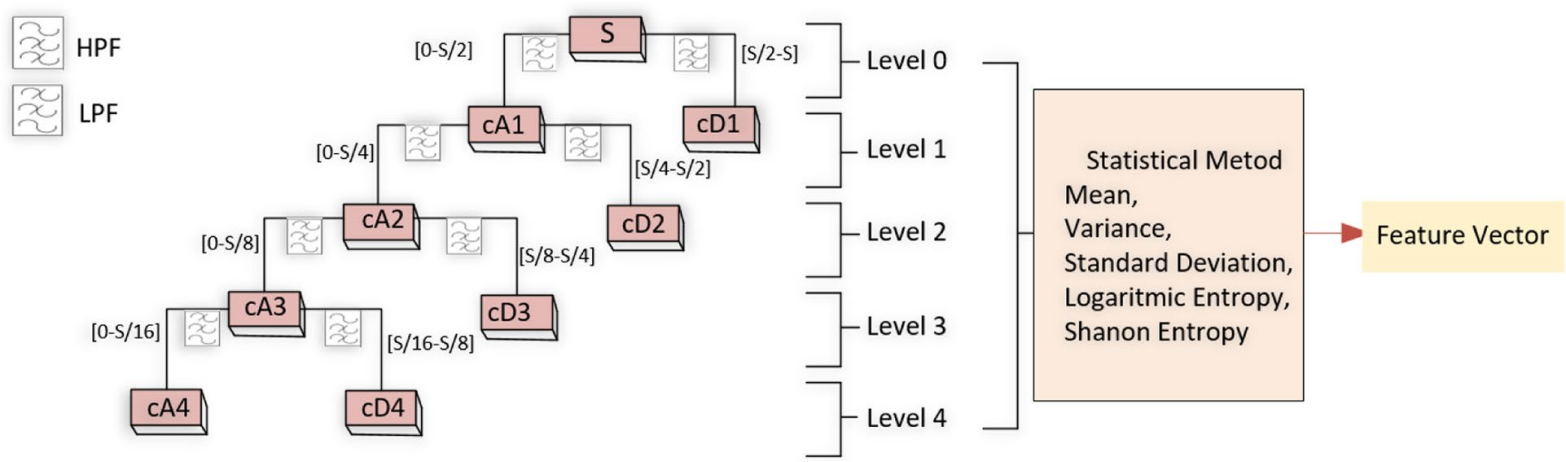
Discrete wavelet transform block diagram

In biomedical signal processing studies, it is known that the frequency domain often provides more information compared to the time domain. While Fourier transformations are commonly used for stationary signals, for signals like EEG, where phase changes over time, time–frequency methods that analyze the scale space effectively are more suitable. Wavelet transformations are preferred to accurately analyze EEG sub-bands and information from the signal [38]. Figure 3 displays the schematic representation of the 4th-level Discrete Wavelet Transform (DWT).

**Fig. 4:**
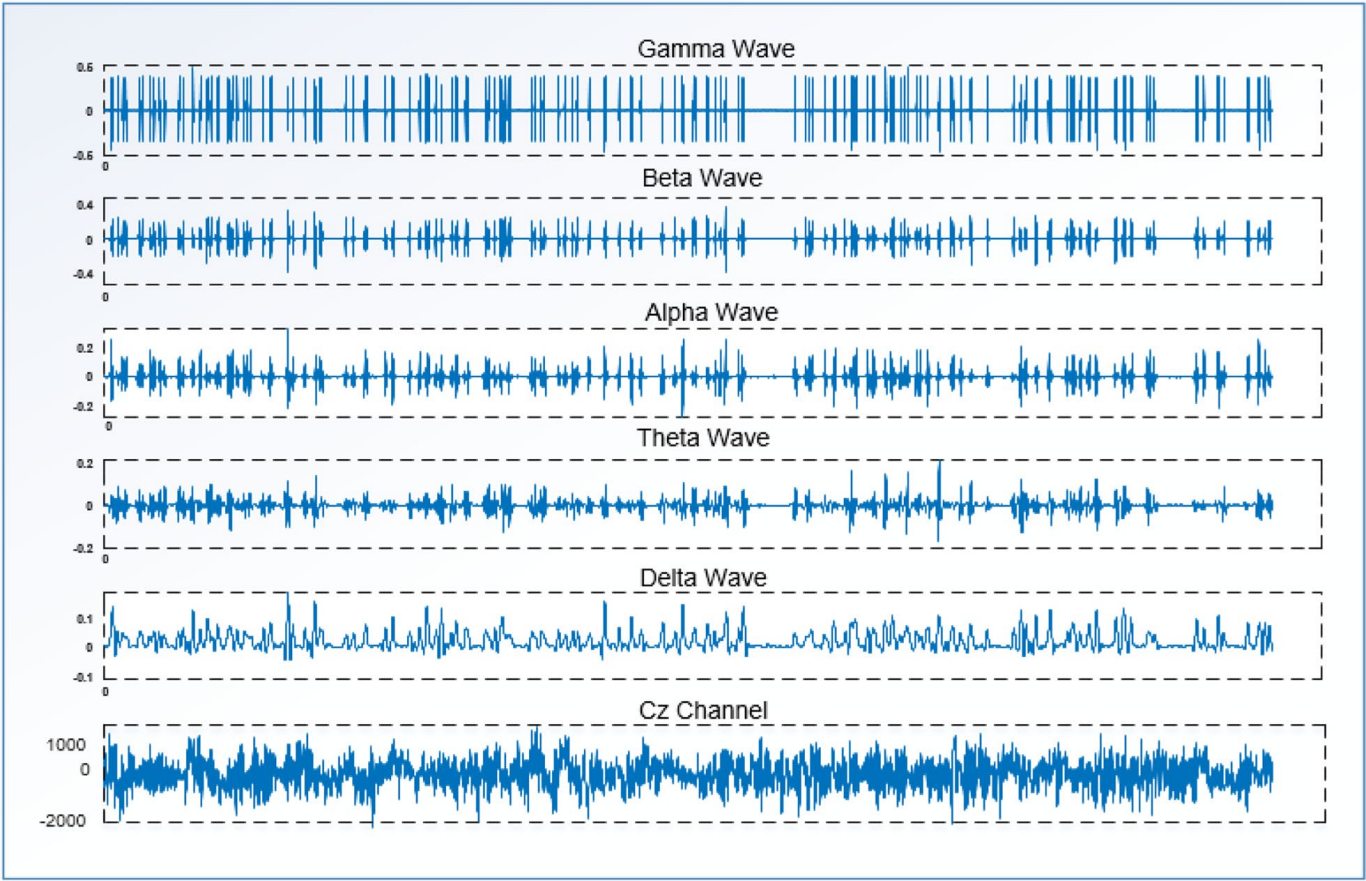
The waves obtained by applying the Discrete wavelet transform to the EEG signal in the Cz channel

In the wavelet transform, detailed (D) and approximate (A) wavelet coefficients are obtained, and these coefficients are used in a linear combination to reconstruct the signal. The approximate coefficient comes from the Low-Pass Filter (LPF), while the detailed coefficient comes from the High-Pass Filter (HPF) [39]. After calculating the coefficients, the approximate coefficient undergoes filtering again and is decomposed level by level until the target signal is reached. Figure 4 shows the result of applying the discrete wavelet transform to the EEG signal obtained from the Cz channel of a patient.

To obtain features from EEG data, the raw EEG signals were first normalized to bring the data within a specific range. After the normalization process, the signals were decomposed into subcomponents using the Daubechies 2 wavelet, which is one of the basic mother wavelet models. The 4th level DWT was applied to the normalized data, resulting in the extraction of Delta (0.5–4 Hz), Theta (4–8 Hz), Alpha (8–15 Hz), Beta (14–30 Hz), and Gamma (30–60 Hz) waves [40].

#### Statistical methods

Following the Discrete Wavelet Transform process, mean, variance, standard deviation, Shannon entropy, and logarithmic entropy methods were applied to the five different wavelet types obtained for each channel. The formulas for the methods used in this stage are provided in Table 1 [7].

**Table 1:** Statistical methods used in the feature extraction stage

**Statistical Method**	
**Method**	**Equation**
Mean	${\mu }_{ti}=1/N{\sum }_{j=i}N {M}_{ij}$
Variance	${{\sigma }_{ti}}^{2}=1/N{\sum }_{j=i}N{\left({M}_{i}-{\mu }_{i}\right)}^{2}$
Standart Deviation	${\sigma }_{ti}={(1/N{\sum }_{j=i}N{\left({M}_{i}-{\mu }_{i}\right)}^{2})}^{1/2}$
Logarithmic Energy Entropy	${H}_{LogEn}\left(x\right)=-{\sum }_{i=0}N-1{\left({log}_{2}\left({p}_{i}\left(x\right)\right)\right)}^{2}$
Shanon Entropy	${H}_{ShanEn}\left(x\right)=-{\sum }_{i=0}N-1{\left({p}_{i}\left(x\right)\right)}^{2}{\left({log}_{2 }\left({p}_{i}\left(x\right)\right)\right)}^{2}$

After applying statistical methods, a dataset with 25 features and one output vector was created for use in the study.

### Dimension reduction

**Fig. 5:**
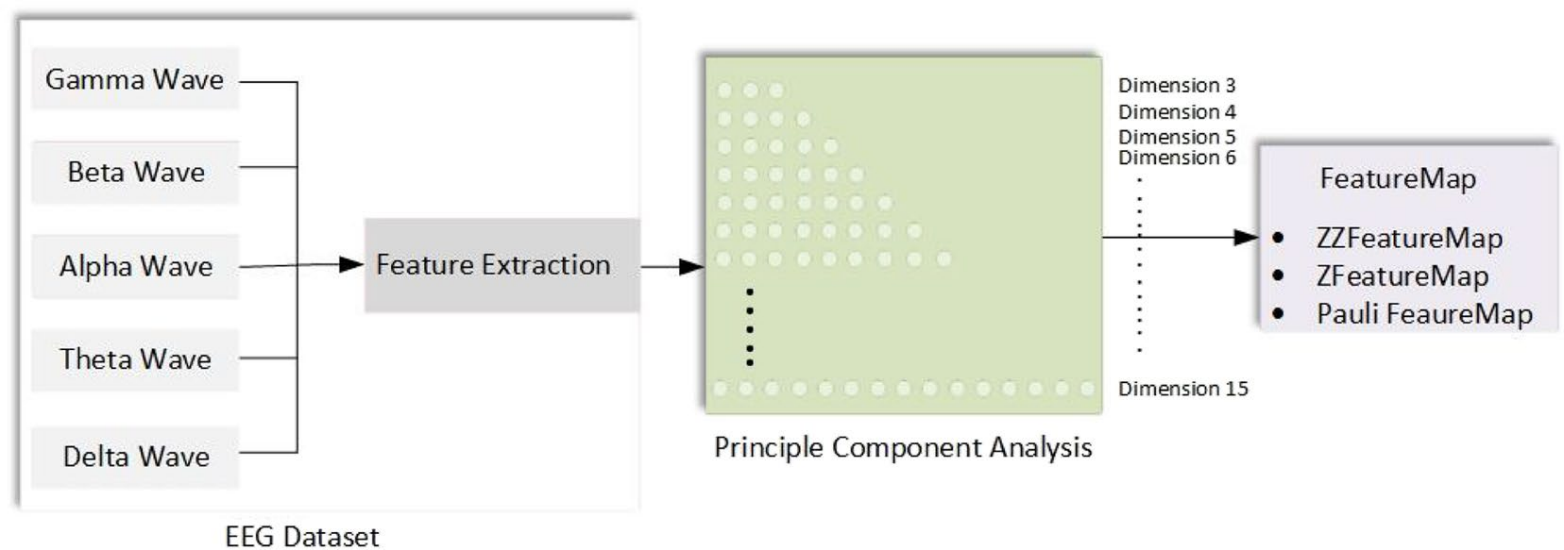
A block diagram illustrating the application of PCA to the dataset

Principle Component Analysis (PCA) is a statistical technique used to reduce the dimensionality of data in large datasets by applying a linear transformation that alters the variables related to unrelated variables. Also known as the Karhunen–Loeve (KL) transformation, this method employs a projection onto an orthogonal subspace to both reduce data dimensionality and create new meaningful variables [41]. Input data is obtained from the principal component with the highest variance. The covariance matrix, utilizing eigenvalues and eigenvectors, allows for the decomposition of brain signals into different components. It reduces the complexity of the classification process but does not guarantee the selection of the most discriminative components [42]. The implementation of PCA is shown in Fig. 5.

Firstly, various preprocessing steps were applied to each EEG sub-band. The data dimension was reduced to values ranging from 3 to 15 using the widely used dimension reduction technique PCA. Feature maps were then applied to each dimension in order to utilize the QSVM algorithm for each dimension.

### Classifiers

The feature vectors created through feature extraction and dimension reduction comprise various features representing the signals. Different machine learning algorithms were used to test their performance on the obtained dataset.

#### Classical machine learning

Machine learning methods are widely used in medical diagnosis systems, where data is mathematically represented and modeled. In these systems, models are trained with data collections created from existing cases. The patterns obtained from trained systems are then analyzed to make predictions based on relationships between data in future cases.

Logistic regression is one of the preferred methods in medical research due to its ability to examine the effects of variables, observe relationships between variables, and analyze the results. Logistic regression analysis, which encompasses various types of variables, relies on past experimental and clinical research as well as statistical analysis methods to determine independent variables. Careful consideration is given to sample size to avoid overfitting. During model development, direct, hierarchical, and statistical approaches are commonly used. The results obtained after simultaneously determining the regression model along with independent variables are interpreted in terms of model fit and individual variables [43].

The K-Nearest Neighbors (K-NN) algorithm is a method based on the similarity between new data and existing data, and it does not require the creation of any model [44]. K-NN is preferred in classification problems because it is easy to implement and resistant to noisy training data. However, achieving the best results with K-NN depends on parameters such as the number of neighbors (k) and the distance metric, making it unclear which distance metric and feature should be used.

A decision tree, also known as a tree-based learning model, is one of the supervised learning models used to solve classification and regression problems. It is used to repeatedly divide a dataset containing a large amount of data into smaller clusters to provide high information gain [45]. Decision tree systems are advantageous for their ability to elucidate complex relationships within data, making them particularly valuable in the medical field where handling incomplete data is common. However, interpreting models can be challenging, especially with small datasets, as overfitting may occur [46].

Random forest algorithm is frequently used in supervised classification and regression problems due to its efficiency and interpretability. This system, in which different decision tree models are used by combining, shows high performance in problems where the number of variables is high [47]. Its fast algorithm and resilience against overfitting have popularized its use for classification.

Naive Bayes classifiers are statistical classifiers that utilize Bayes' theorem. This algorithm assesses the probabilities of current events to predict the probabilities of future events [48].

SVM is a machine learning algorithm based on a supervised learning technique. It is a method based on statistical learning theory used to classify both non-linear and multi-class data. In this method, data is separated by a hyperplane. Decision functions obtained as a result of training the data are used to label the test data [49].

#### Quantum support vector machine

Quantum technology enables the solution of problems that are difficult to solve on traditional computers by using the properties and events discovered in the field of quantum physics. Quantum machine learning is a versatile discipline that brings together the fields of quantum physics and classical machine learning. In this field, quantum variations of machine learning algorithms are created by taking advantage of the power of quantum computing. Contrary to the binary system existing in traditional computers, in quantum systems data can be in more than one state (superposition) at the same time. This is represented by quantum bits called *qubits*, which are the basic unit of quantum computing. When qubits are in superposition, more than one situation can be evaluated at the same time, so the running times of the algorithms can be accelerated.

QSVM, is a type of classifier that uses the quantum state space as a feature space for the analysis of classically provided data. It is a transformed version of the SVM algorithm using quantum computing features [50]. For the algorithm to be used, classical data needs to be mapped to the quantum state in a nonlinear way. Mapping to quantum states is achieved by encoding the features in qubit form. To perform this operation, quantum maps are applied to the initial state. As a result, a kernel quantum circuit is created using this method. This allows data to be classified in high-dimensional spaces. Each quantum map results in different kernels, thus affecting the classification performance. In the final step, a non-linear kernel is used to create a hyperplane, and the data is classified. The feature maps used in the study, prepared with 3 qubits and a single repetition, are shown in Appendix 1.

At this stage, 13 different feature maps (as depicted in Appendix 1) were applied to convert classical data into quantum state. Among these maps, there is two instances of ZZ feature maps, respectively with linear a full entanglement. FeatureMaps were used so that the number of repetitions of the circuit was two.

In general, when examining the feature maps, it can be observed that in all maps, the Hadamard gate is applied to all qubits, initially putting the qubits in a superposition state. Superposition is defined as a qubit being in multiple states simultaneously. In ZZ feature maps, the option of whether the data will be circulated with the following qubits or with all the following qubits is determined using the linear or full feature. In all maps except Pauli X, Y, and Z feature maps, the CNOT gates are used, which perform an operation known as quantum entanglement, where the value of one qubit is created based on the value of another qubit. The P function present in all feature maps represents the interaction between qubits. The Rx gate rotates the qubit at a certain angle on the x-axis. The created feature maps serve as kernels in QSVM [51]. Each feature map is independent of each other and is created using different gates.

In the final stage, a quantum kernel transformation is used to create a nonlinear high-dimensional feature space, and a classification process is performed by creating a hyperplane to separate the labeled data used by a classical SVM [52].

In all analyses, the IBM Quantum Lab environment was used to produce results independent of computer hardware. QasmSimulator, which allows the use of 32 qubits, was preferred for the analysis of the QSVM algorithm.

## Experimental results

In this section, analyses using EEG signals for the diagnosis of Schizophrenia are shared. The performances of the analyses performed using classical machine learning algorithms and QSVM algorithms were examined according to speed and accuracy values. In this study, the data set used was divided according to 70% training and 30% testing ratios. While segmenting the data, the random sample selection method was used to ensure that reliable results could be obtained on the training and test data of the model.

In the study, different numbers of qubits and different feature maps were used for the quantum algorithm. EEG data were prepared for analysis by undergoing certain preprocessing. Dimension reduction was performed on the prepared dataset. The algorithms used in the study were prepared with the Python programming language. To apply quantum machine learning methods, the Qiskit library and various libraries under the Qiskit library were used. Classical and quantum machine learning techniques were applied in the IBM Quantum Lab environment according to the number of each feature. All analyses performed in the study were run online, using the capability of IBM Quantum cloud for free users.

### Results obtained from classical machine learning algorithms

**Fig. 6:**
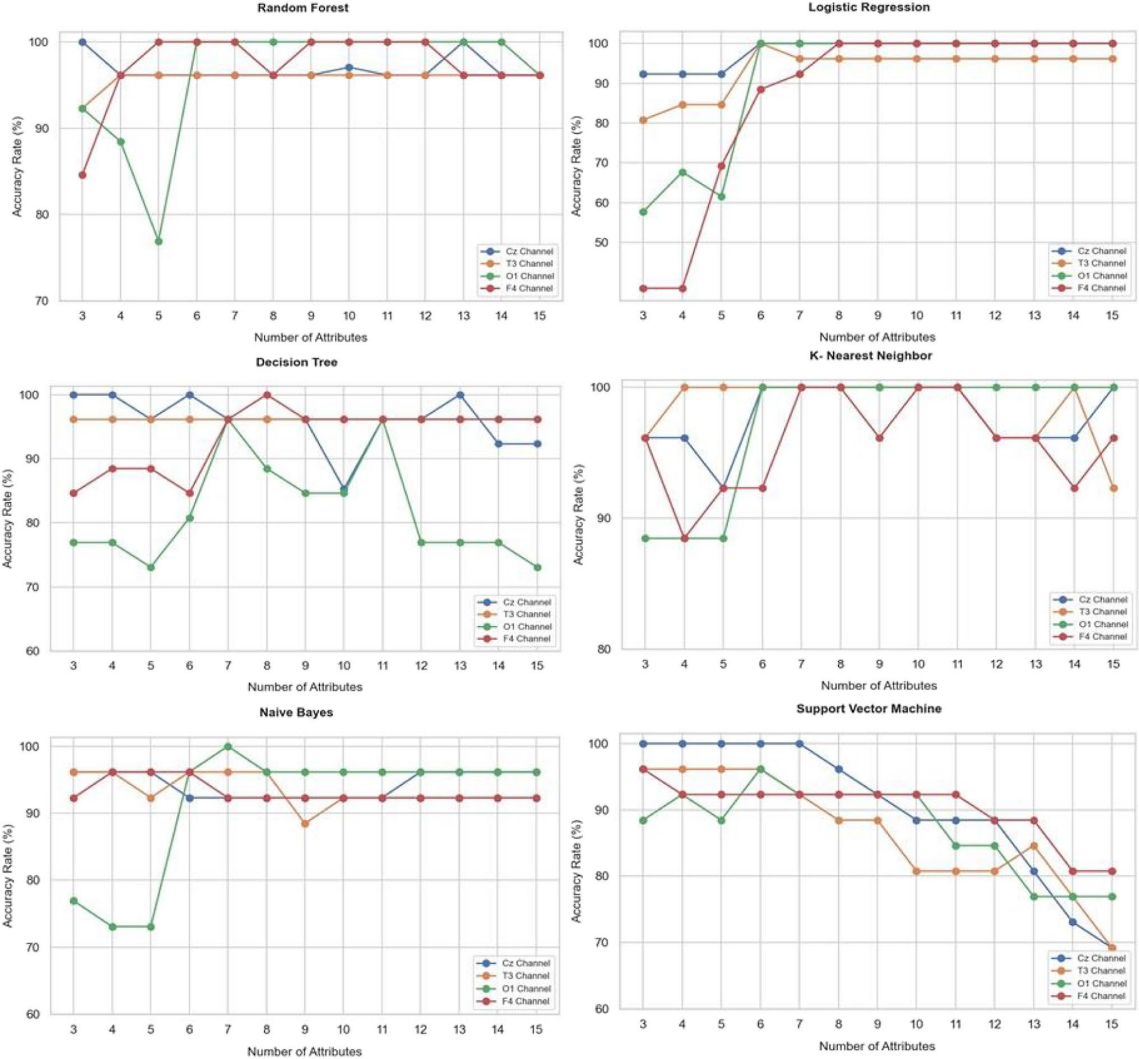
The performance graph of classical machine learning algorithms for 4 channels

In the study, the data set prepared using EEG signals was converted into 12 different features using PCA. Random Forest, Logistic Regression, Decision Tree, K Nearest Neighbor, Naive Bayes, and Support Vector Machine Algorithms were applied for each attribute value, starting from 3 to 15 attributes. These operations were carried out for 4 channels. Graphs showing the accuracy values obtained according to the different machine learning methods applied to each channel separately and the number of attributes are shown in Fig. 6.

When the graphs in Fig. 6 are examined, it can be observed that the best performance was obtained from the Central region's Cz and the temporal region's T3 channels. Therefore, Table 2 presents the performance values based on classical methods obtained from the number of features extracted from the Cz and T3 channels.

**Table 2:** Accuracy rates obtained from the Cz and T3 channels for classical classifiers

	**Cz Channel**						**T3 Channel**					
**Number of Attributes**	**Methods and Accuracy Values (%)**											
	RF	LR	DT	kNN	NB	SVM	RF	LR	DT	kNN	NB	SVM
3	100	92.31	100	96.15	96.15	100	92.31	80.77	96.15	96.15	96.15	96.15
4	96.15	92.31	100	96.15	96.15	100	96.15	84.62	96.15	100	96.15	96.15
5	96.15	92.31	96.15	92.31	96.15	100	96.15	84.62	96.15	100	92.30	96.15
6	96.15	100	100	100	92.30	100	96.15	100	96.15	100	96.15	96.15
7	96.15	100	96.15	100	92.30	100	96.15	96.15	96.15	100	96.15	92.31
8	96.15	100	96.15	100	92.30	96.15	96.15	96.15	96.15	100	96.15	88.46
9	96.15	100	96.15	100	92.30	92.31	96.15	96.15	96.15	100	88.46	88.46
10	97.06	100	85.29	100	92.30	88.46	96.15	96.15	96.15	100	92.30	80.77
11	96.15	100	96.15	100	92.30	88.46	96.15	96.15	96.15	100	92.30	80.77
12	96.15	100	96.15	96.15	96.15	88.46	96.15	96.15	96.15	96.15	92.30	80.77
13	100	100	100	96.15	96.15	80.77	96.15	96.15	96.15	96.15	92.30	84.62
14	96.15	100	92.31	96.15	96.15	73.08	96.15	96.15	96.15	100	92.30	76.92
15	96.15	100	92.31	100	96.15	69.23	96.15	96.15	96.15	92.31	92.30	69.23

In the Cz channel, the highest accuracy rate, mostly at 100% for feature counts between 6–15, was obtained from the Logistic Regression algorithm. In the T3 channel, the best performance, with 100% accuracy, was achieved for feature counts between 4–11 using the K Nearest Neighbor algorithm. Overall, for the 4 channels, the Logistic Regression algorithm has shown higher performance compared to other algorithms. In the SVM algorithm, it can be observed that accuracy decreases as the number of features increases. The results for classification performance metrics obtained for 5 features from Cz and T3 channels are presented in Table 3.

**Table 3:** Classification performance metric results obtained for 5 qubits from Cz and T3 channels

	**Cz Channel**						**T3 Channel**					
**Classification Performance Metrics**	**Methods**											
	RF	LR	DT	kNN	NB	SVM	RF	LR	DT	kNN	NB	SVM
Specificity	1.0	0.81	1.0	0.81	0.90	1.0	0.90	0.69	0.90	1.0	0.81	0.90
Precision	1.0	0.88	1.0	0.88	0.94	1.0	0.94	0.76	0.94	1.0	0.88	0.94
Sensitivity	0.94	1.0	0.94	1.0	1.0	1.0	1.0	1.0	1.0	1.0	1.0	1.0
F1-Score	0.96	0.93	0.97	0.93	0.96	1.0	0.96	0.86	0.96	1.0	0.93	0.96

In both channels, RF and SVM methods generally exhibit high specificity values, while variability is observed in LR and DT methods. RF, SVM, and DT methods have high precision values. Overall, high sensitivity values are observed for all methods. High performance is evident for the kNN algorithm in this classification task.

### Results obtained from QSVM algorithm

A quantum-based machine learning algorithm, QSVM, was used in this study. To use this method, the bits were converted into qubits using feature maps. During this process, 13 different feature maps were used with 2 repetitions. The number of qubits varied between 3 and 15 while applying the feature maps. The transformation to qubit form was performed separately for each feature count. When classical machine learning algorithms were applied, the highest accuracy values were obtained from the Cz and T3 channels among the four channels. The accuracy values obtained from the QSVM algorithm for the Cz channel are provided in Table 4.

**Table 4:** Accuracy rates of QSVM when different FeatureMaps are applied to Cz channel EEG signals

**Qubit Number**	**ZZ** **FeatureMap**		**Pauli FeatureMap**										
	Linear	Full	Z	X	Y	XX	XZ	ZX	YZ	ZY	Z, YY	Z, XX	Z, Y, ZZ
3	100	100	100	100	34.61	92.30	76.92	88.46	92.30	80.76	69.23	88.46	73.07
4	100	92.30	100	100	34.61	80.76	80.76	84.61	96.15	80.76	73.07	92.30	73.07
5	92.30	76.92	100	100	34.61	92.30	84.61	80.76	84.61	76.92	76.92	80.76	65.38
6	92.30	73.07	100	100	34.61	73.07	69.23	76.92	84.61	73.07	61.53	73.07	65.38
7	84.61	65.38	100	100	34.61	96.15	61.53	80.76	65.38	65.38	53.84	61.53	46.15
8	80.76	53.84	100	100	34.61	84.61	46.15	73.07	57.69	57.69	42.30	53.84	38.46
9	76.92	50.00	100	100	34.61	80.76	38.46	57.69	50.00	53.84	34.61	50.00	38.46
10	76.92	38.46	100	100	34.61	91.17	55.88	73.52	61.76	58.82	50.00	61.76	50.00
11	69.23	34.61	100	100	34.61	80.76	34.61	34.61	34.61	34.61	34.61	34.61	34.61
12	61.53	34.61	100	100	34.61	57.69	34.61	34.61	34.61	34.61	34.61	34.61	34.61
13	57.69	34.61	96.15	100	34.61	73.07	34.61	34.61	34.61	34.61	34.61	34.61	34.61
14	46.15	34.61	96.15	100	34.61	42.30	34.61	34.61	34.61	34.61	34.61	34.61	34.61
15	42.30	34.61	96.15	100	34.61	46.15	34.61	34.61	34.61	34.61	34.61	34.61	34.61

When examining the table, it can be observed that ZZ features maps provide better accuracy when the entanglement is linear rather than full. Except for the Z and X feature map, the accuracy rate of QSVM is very low for 11–15 qubits. The best performance is achieved for 4 qubits. X feature map achieved 100% performance for all qubit values. From 5 qubits onwards, a decline in performance is observed across various feature maps. Table 5 presents the values obtained for the Cz channel for 5 qubits, based on other classification performance metrics.

**Table 5:** The results of classification performance metrics obtained from the Cz channel for 5 qubits

**Classification Performance Metrics**	**ZZ** **FeatureMap**		**Pauli FeatureMap**										
	Linear	Full	Z	X	Y	XX	XZ	ZX	YZ	ZY	Z, YY	Z, XX	Z, Y, ZZ
Specificity	0.81	0.61	1.0	1.0	0.34	0.81	0.72	0.64	0.72	0.61	0.63	0.64	0.50
Precision	0.88	0.70	1.0	1.0	0.0	0.88	0.82	0.70	0.82	0.70	0.76	0.70	0.52
Sensitivity	1.00	0.92	1.0	1.0	0.0	1.0	0.93	1.00	0.93	0.92	0.86	1.00	0.90
F1-Score	0.93	0.79	1.0	1.0	0.0	0.93	0.87	0.82	0.87	0.79	0.81	0.82	0.66

When examining the performance values obtained for 5 qubits in the Cz channel, it is observed that the 'X' and 'Z' feature maps demonstrate excellent performance across various metrics, while the 'Y' feature map exhibits lower performance. Table 6 presents the results obtained from the utilization of different feature maps in the T3 channel, using the QSVM algorithm.

**Table 6:** QSVM accuracy rates obtained when applying different FeatureMaps to T3 channel EEG signals

**Qubit Number**	**ZZ** **FeatureMap**		**Pauli FeatureMap**										
	Linear	Full	Z	X	Y	XX	XZ	ZX	YZ	ZY	Z, YY	Z, XX	Z, Y, ZZ
3	92.30	92.30	96.15	96.15	34.61	92.30	84.61	92.30	88.46	88.46	88.46	88.46	84.61
4	92.30	92.30	96.15	100	34.61	80.76	84.61	92.30	88.46	88.46	92.30	84.61	84.61
5	92.30	80.76	100	100	34.61	100	80.76	84.61	88.46	84.61	84.61	84.61	76.92
6	92.30	76.92	100	100	34.61	84.61	73.07	80.76	80.76	76.92	69.23	69.23	76.92
7	88.46	73.07	100	100	34.61	92.30	65.38	76.92	73.07	69.23	73.07	69.23	61.53
8	88.46	65.38	100	100	34.61	88.46	65.38	65.38	65.38	65.38	53.84	65.38	46.15
9	80.76	53.84	100	100	34.61	88.46	46.15	57.69	50.0	42.30	38.46	42.30	34.61
10	76.92	38.46	100	100	34.61	69.23	34.61	38.46	38.46	34.61	34.61	34.61	34.61
11	73.07	34.61	100	100	34.61	80.76	34.61	38.46	34.61	34.61	34.61	34.61	34.61
12	61.53	34.61	100	100	34.61	46.15	34.61	38.46	34.61	34.61	34.61	34.61	34.61
13	57.69	34.61	96.15	100	34.61	69.23	34.61	34.61	34.61	34.61	34.61	34.61	34.61
14	38.46	34.61	96.15	96.15	34.61	38.46	34.61	34.61	34.61	34.61	34.61	34.61	34.61
15	38.46	34.61	96.15	96.15	34.61	42.30	34.61	34.61	34.61	34.61	34.61	34.61	34.61

When looking at the results obtained from EEG signals taken from the T3 channel, it is observed that the best performance was achieved for 2-repetition feature maps with Z and X feature maps. In the case of other feature maps, the accuracy rate decreases as the number of qubits increases. Table 7 provides the results of the performance metrics obtained for the T3 channel.

**Table 7:** The results of classification performance metrics obtained from the T3 channel for 5 qubits

**Classification Performance Metrics**	**ZZ** **FeatureMap**		**Pauli FeatureMap**										
	Linear	Full	Z	X	Y	XX	XZ	ZX	YZ	ZY	Z, YY	Z, XX	Z, Y, ZZ
Specificity	0.81	0.64	1.0	1.0	0.34	1.0	0.70	0.69	0.75	0.69	0.69	0.69	0.60
Precision	0.88	0.70	1.0	1.0	0.0	1.0	0.82	0.76	0.82	0.76	0.76	0.76	0.64
Sensitivity	1.00	1.00	1.0	1.0	0.0	1.0	0.87	1.0	1.0	1.0	1.0	1.0	1.0
F1-Score	0.93	0.82	1.0	1.0	0.0	1.0	0.84	0.86	0.90	0.86	0.86	0.86	0.78

Below, the graphical representation of the results obtained from the channels used for Z and Pauli X feature map, which exhibited the best performance for both channels, is provided.

**Fig. 7:**
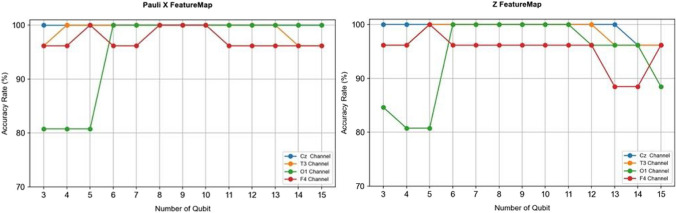
The performance graph of the QSVM algorithm using Pauli X and Pauli Z Feature Maps for 4 channels

According to Fig. 7, it can be observed that the best performance is achieved with Pauli X and Z feature maps using the Cz and T3 channels. In these feature maps, the number of qubits did not have any impact on the analysis results. However, for the O1 and F4 channels, changes in the number of qubits have affected the accuracy rate.

## Discussion

In this study, various classical and quantum-based machine learning algorithms were applied to EEG data sets obtained from both schizophrenic patients and healthy individuals for the diagnosis of schizophrenia. According to the results obtained using classical machine learning methods, it was found that Logistic Regression performed best for the Cz channel, while k-Nearest Neighbors (kNN) was the top-performing algorithm for the T3 channel. Logistic Regression achieved 100% accuracy for 6–15 features in the Cz channel, and kNN yielded the highest performance for 2–11 features in the T3 channel. As the number of features increased for all four channels, the performance of the Support Vector Machine (SVM) algorithm decreased. Considering that the Quantum Support Vector Machine (QSVM) algorithm is an adaptation of the SVM algorithm for quantum computers, this decline in performance is noteworthy. When compared to classical machine learning methods, the QSVM algorithm delivers similar, and sometimes slightly better results in cases where specific feature maps are used namely X and Z feature maps.

When examining the results obtained with the QSVM algorithm, it is evident that the feature maps have a significant impact on the performance of quantum machine learning methods. According to the results obtained for each channel, the QSVM algorithm performs better for all feature maps when the number of qubits is low. Except for the Z and X feature maps, the other maps show a decrease in performance starting from 5 qubits, and a significant drop in accuracy is observed from 10 qubits onwards. The increase in the number of qubits and the use of CNOT and Rx gates contribute to the complexity of the circuits, leading to this outcome. When considering all the circuits, only the circuits that include Hadamard gates and the P function are not affected by the increase in the number of qubits in terms of performance. In cases where CNOT gates are used, the complexity of the circuit increases, the accuracy decreases, and the algorithm's execution time also increases since the state of one qubit affects the state of the target qubit in the circuit. When the number of qubits is increased, it has been observed that the ZZ feature map entanglement, when selected linearly, results in a smaller decrease in accuracy compared to when the full feature is selected. For the Y feature map, when the QSVM algorithm is run, it was observed that the results were quite poor for all qubits from 3 to 15. When the circuit repetition was increased for this feature map, it was found that the results improved. However, in this study, all the feature maps had their parameters fixed, so this aspect was not included in the analysis. It was concluded that running the Y feature map with 2 repetitions may not be suitable for this dataset.

The methods for classical and quantum machine learning were compared in terms of accuracy rates and algorithm execution times in the created model. All analyses were conducted in the IBM Quantum Lab environment, allowing for the comparison of results. It was found that classical machine learning algorithms generally completed within 0.01 s. However, the execution time of the QSVM algorithm varied depending on the used feature map. In general, when the number of qubits was low, the analyses were completed in less than 1 min. However, as the number of qubits increased, the complexity of the circuits resulted in significantly longer execution times. When comparing feature maps, Pauli X and Z feature maps were found to have the shortest execution times for the QSVM algorithm. The use of a quantum simulator environment instead of a quantum computer had a negative impact on the study's execution time. The queuing of operations in quantum computers and the limitation, at the time of writing, of a maximum of 7 qubits in publicly accessible quantum computers led to the preference for quantum simulators over real quantum computers for analyses.

Table 8 lists the studies conducted on the data set used in the article. The table presents the preprocessing and classification algorithms used in these studies. They carried out their analysis using classical machine learning algorithms and deep learning models. Differences in the preprocessing stages and methods used in the literature cause the obtained performances to vary. In this study, in addition to the DWT method, various statistical techniques were applied to the data set during the pre-processing stage and the data set was tried to be made most efficient. Unlike other studies in the literature, the analyses were carried out on a single channel. Due to the limited use of qubits offered by quantum platforms, the size of the data set was gradually reduced with the PCA algorithm. The new data set obtained by applying all these processes was subjected to an experimental process with QSVM and classical classification algorithms. When the analysis results were examined, it was determined by the authors that the performance of the proposed method was higher compared to the previous studies.

**Table 8:** Performance comparisons with some studies related to the dataset used in the article

**Researchers**	**Preprocessing**	**Methods**	**Accuracy (%)**	**Sensitivity** **(%)**	**Specificity** **(%)**
Piryatinska et al. (2017) [53]	ϵ-complexity function	SVM, RF	89.38, 85.30	88.6	82.6
Naira and Alamo (2019) [54]	Pearson Correlation Coefficient (PCC)	CNN	90.00	90.00	90.00
Bougou et al. (2019) [55]	Butterworth filter Connectivity Analysis	RF	82.36	-	-
Phang et al. (2020) [56]	Time-domain VAR coefficients, Frequency-domain PDC, Topological-based CN measures	MDC-CNN, SVM	93.06 85.83	95.00 87.50	91.11 88.89
Singh et al. (2020) [57]	Butterworth bandpass Filter, Hjorth descriptors	CNN-SF, LSTM	94.08 76.78	92.70 80.72	95.31 73.18
Phang et al. (2020) [58]	VAR, PDC, CN	SVM, CNN, RNN	90.37 91.69 77.50	91.11 91.11 86.67	89.64 92.50 66.79
Aslan and Akın (2020) [59]	Short-time Fourier Transform (STFT)	CNN (VGG-16)	95.00	95.00	95.00
Rajesh et al. (2021) [60]	Symmetrically Weighted Local Binary Pattern: SLBP	Logitboost	91.66	89.74	93.33
Khodabakhsh et al. (2021) [61]	Brain Functional Connectivity (FC)	MDC-CNN FC-UNET	90.44 94.11	97.78 91.66	81.79 100.0
Supakar et al. (2022) [62]	Random Projection (Dimensionality reduction)	RNN- LSTM	98.00	98.00	98.00
Xin et al. (2022) [63]	Normal and İmproved high-order functional connectivity matrices, Finite Impulse Response (FIR).	SVM	94.05	95.56	92.31
Sairamya et al. (2022) [64]	Discrete wavelet transform, Relaxed Local Neighbour Difference Pattern (RLNDiP).	YSA	100.0	100.0	100.0
Aslan and Akın M. (2022) [65]	Hilbert Huang Transform (HHT), Hilbert Spectrum (HS).	VGG16, XCeption, ResNet152, InceptionV3.	96.00 95.00 95.00 87.50	96.00 95.50 95.50 88.50	96.00 97.50 94.50 92.00
Sobahi et al. (2022) [66]	Daubechies 4th order wavelet function, Local Binary Pattern (LBP).	CNN	97.70	97.80	97.60
Alves et al. (2022) [67]	Granger causality test, Pearson’s correlation coefficient, Spearman’s correlation measures.	CNN	52.00 57.00 62.00	73.00 100.0 100.0	- - -
Kumar et al. (2023) [68]	Symmetrically Weighted-Local Binary Patterns (SLBP), Histogram of Local Variance (HLV)	AdaBoost	92.85	97.80	87.20
Balasubramanyan et al. (2023) [69]	Butterworth band pass filter, WICA, Relief algorithm.	Hybrid Grey Wolf-Bat Algorithm- ANFIS	99.51	95.87	-
Proposed Method	DWT, Statistical Methods	QSVM (Pauli X, Pauli Z)	100.0	100.0	100.0

The main advantages of this study can be summarized as follows. To the best of the authors' knowledge, it is the first quantum machine learning study in the field of schizophrenia. The method demonstrates encouraging results, being able to generalize from the data, using only a limited number of components. For this reason, it forms the basis for future studies in the field of neuroscience. Even if not all channels of the data set were used in the study, the method showed better performance compared to studies in the literature. Although there are various experimental studies using the QSVM algorithm, no study has been found that investigated the impact of the feature maps on the EEG data, as it was implemented in this study. This analysis shows that quantum circuits and gates greatly affect the performance obtained from the QSVM algorithm. It has been proven that the performance of QSVM can be maximized with correctly selected feature maps.

In addition to the stated advantages of the study, the method we used has various limitations since quantum is a new and rapidly evolving field. The main limitations experienced in the study can be summarized as follows. The use of a quantum simulator environment instead of a real quantum computer had a negative impact on the duration of the study. Real platforms could not be used due to the queuing of transactions in quantum computers and the qubit limitations of quantum computers that everyone can access. One of the important contributions of the study is to demonstrate the impact of the number of qubits on classification performance, which is why the number of qubits used in the study was set between 3 and 15. Operations carried out on 13 feature maps with a limited number of qubits did not involve the use of real machines for this reason. As a reminder, and at the time of writing, the number of available qubits on publicly available real quantum computer is only seven qubits.

In the future, research will be conducted to examine the performances of different quantum machine learning algorithms in the literature. It is planned to carry out studies to optimize and improve the parameters of existing methods (e.g., number of repetition) and to test on different simulators, such as the ones based on density matrix and state vector, which are reputed to be quick and efficient. If access to real machines with a high number of qubits is provided, the studies to be carried out will be analyzed on real quantum computers.

Another question that naturally arise is whether quantum machine learning can provide an advantage over classical computing, by replacing or complement existing algorithms in the domain of healthcare. A first direction to answer this question, is to evaluate the performance of quantum machine learning in situation where classical computing fails. A second research direction is to compare the predictions made by quantum and classical pipelines. A similar approach was investigated in [70] where the authors trained a meta-classifier on a subset of the data where the quantum and classical classifiers provided different predictions.

## Conclusions

The main goal of this study is to investigate the effectiveness of machine learning algorithms in the early diagnosis and initiation of the treatment process for psychological disorders such as schizophrenia. To achieve this goal, four channels to be used in the model were initially determined. EEG signals were divided into 5 sub-bands by applying discrete wavelet transform to these four channels. A dataset for analysis was obtained by applying specific statistical techniques to each subband. PCA was applied to reduce the dimensionality of the data. To use the data in quantum systems, they were converted to qubit form. For this purpose, the effects of various feature maps on the QSVM algorithm were examined. Classical machine-learning methods were also applied to the dataset. As a result of the analyses, despite being a newly emerging technology, it was observed that quantum machine learning achieved state-of-the-art and sometimes slightly better results. In the study, the best results were obtained using a limited number of qubits. Datasets with a higher signal-to-noise ratio might require more qubits. However, this is generally promising for EEG classification. Experimental study results indicate the potential of quantum technologies for healthcare. In the domain of schizophrenia, our work suggests that quantum machine learning can help with the diagnosis of schizophrenia, while using a limited number of components as compared to the classical approaches. Additional work is required to evaluate if quantum and classical approaches exploit different relationship between the data, and if there is an interest in using a hybrid method for the detection of schizophrenia. The results obtained for different repeat parameters of feature maps will be also evaluated in future studies. New studies will be conducted using real quantum computers and different simulator environments to explore the accuracy and execution times of various quantum-based algorithms.

## Appendix

### Appendix 1. Quantum Feature Maps used in the study, obtained with Qiskit 0.44.3 (IBM, Armonk, NY, the US), a Python library for quantum computing. In the schema, PauliFeatureMap, ZZFeatureMap and ZFeatureMap refer to the Qiskit implementation of the feature maps


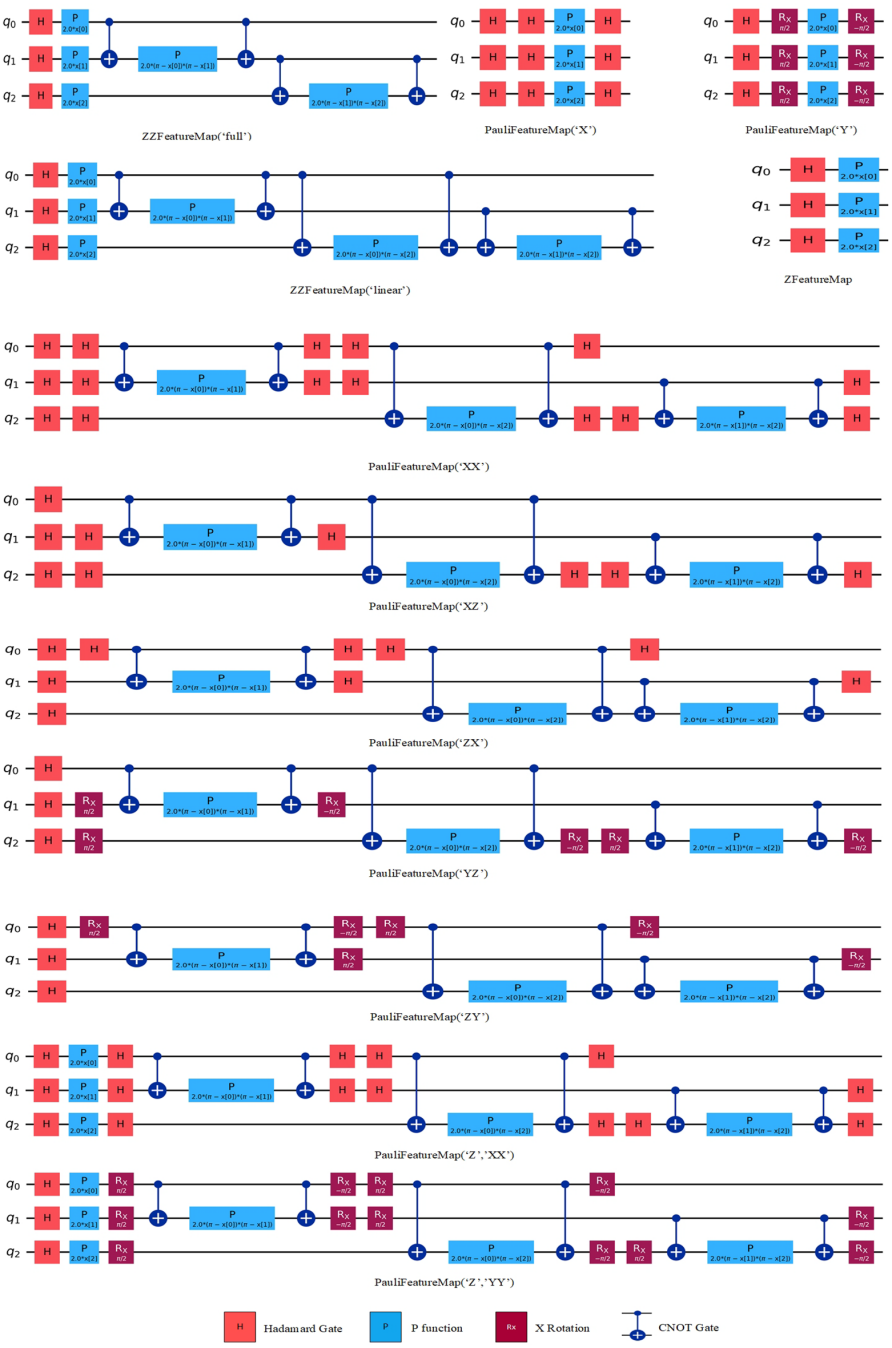


## Data Availability

In the study, an EEG dataset of healthy adolescents and adolescents with symptoms of schizophrenia, which is located in the Neurophysiology and Neuro-Computer Interfaces Laboratory, was used. All the data can be accessed publicly at http://brain.bio.msu.ru/eeg_schizophrenia.htm, Accessed on: 11.02.2022. No datasets were generated or analysed during the current study.
